# Mild ovarian stimulation with letrozole plus fixed dose human menopausal gonadotropin prior to IVF/ICSI for infertile non-obese women with polycystic ovarian syndrome being pre-treated with metformin: a pilot study

**DOI:** 10.1186/s12958-018-0405-3

**Published:** 2018-09-14

**Authors:** Giuseppe D’Amato, Anna Maria Caringella, Antonio Stanziano, Clementina Cantatore, Simone Palini, Ettore Caroppo

**Affiliations:** 1 Asl Bari, Department of Maternal and Child Health, Reproductive and IVF Unit, Conversano, BA Italy; 2 ASL Bari, PTA “F Jaia”, Fisiopatologia della Riproduzione Umana e P.M.A, via de Amicis 30, 70014 Conversano, BA Italy

**Keywords:** Letrozole, Human menopausal gonadotropin, Polycystic ovarian syndrome, Mild ovarian stimulation, IVF, Estradiol level, OHSS

## Abstract

**Background:**

Letrozole is widely employed as ovulation induction agent in women with PCOS, but its use in mild stimulation (MS) protocols for IVF is limited. Aim of the present study was to evaluate the feasibility of a MS protocol with letrozole plus hMG in non-obese PCOS women undergoing IVF after a metformin pre-treatment.

**Methods:**

We retrospectively evaluated the data of 125 non-obese PCOS undergoing MS with letrozole plus hMG, 150 IU as starting dose, (group 1, *N* = 80) compared to those undergoing a conventional IVF stimulation protocols (CS) (group 2, *N* = 45) prior to IVF. All patients had received metformin extended release 1200–2000 mg daily for three to six months before IVF. GnRH antagonist was administered in both groups when the leading follicles reached 14 mm.

**Results:**

Both groups were comparable for age, BMI and ovarian reserve markers. Both groups showed lower than expected AFC and AMH values as a consequence of metformin pre-treatment. Letrozole-treated patients required a significantly lower amount of gonadotropins units (*p* < 0.0001), and showed significantly lower day 5, day 8 and hCG day E2 levels compared to patients undergoing the CS protocol (*p* < 0.0001, *p* < 0.0001 and *p* = 0.001 respectively). The oocyte yield, in terms of total (6, IQR 3, vs 6, IQR 4 respectively,) and MII oocytes (5, IQR 3, vs 5, IQR 3, respectively) number, did not differ among groups; the number of total (3, IQR 2, vs 3, IQR 1 respectively) and good quality embryos (2, IQR1 vs 2, IQR 1,5 respectively) obtained was comparable as well in the two groups. The number of fresh transfers was significantly higher in group 1 compared to group 2 (80% vs 60%, *p* = 0.016). A trend for higher cumulative clinical pregnancy rate was found in women undergoing MS compared to CS (42.5%vs 24,4%, *p* = 0.044), but the study was not powered to detect this difference.

**Conclusions:**

The present study suggests that the use of letrozole as adjuvant treatment to MS protocols for IVF may be an effective alternative to CS protocols for non-obese PCOS patients pre-treated with metformin, as it provides comparable IVF outcome without requiring high FSH dose, and avoiding supraphysiological estradiol levels.

## Background

Mild ovarian stimulation (MS) for in vitro fertilization (IVF) consists in the administration of follicle stimulating hormone (FSH) or human menopausal gonadotropin (hMG) at lower dose and/or for a shorter duration, in a gonadotropin releasing hormone (GnRH)-antagonist co-treated cycle, with or without the concomitant use of oral compounds, antiestrogens or aromatase inhibitors (AI), with the aim of collecting fewer oocytes [1]. It is intended to offer a safer, cost-effective, patient-friendly protocol in which the risks of treatment are minimized [2], and in which the benefits are grossly comparable to those provided by conventional IVF stimulation protocols (CS). Relevant to this point, studies have demonstrated that increasing the FSH may not improve the IVF outcome, since there is no direct relationship between FSH dose and embryo quality [3], and the number of euploid embryos don’t differ when MS or conventional ovarian stimulation protocols are employed [4].

Letrozole, together with clomiphene citrate, is one of the mostly used oral compounds in MS protocols, and is preferred to anastrazole due to its higher in vitro inhibitory effect on the enzyme aromatase and reduction of estrogen plasma levels [5]. Letrozole has been also utilized in ovulation induction cycles in clomiphene-resistant women with PCOS, without impacting the endometrial thickness despite the reduced total and per follicle estrogen levels on the day of hCG administration [6]; when compared to clomiphene citrate, it improves pregnancy and live birth rates in subfertile women with anovulatory PCOS [7].

Despite being promising as ovulation induction agent, letrozole has drawn limited attention as adjuvant treatment to MS protocols for IVF/ICSI cycles, as it doesn’t appear to significantly improve pregnancy rates compared to the standard controlled ovarian stimulation protocols [8, 9]. As far as we could know, no study has been drawn to evaluate its use in infertile women with polycystic ovary syndrome undergoing IVF.

Aim of this pilot study was, therefore, to evaluate the feasibility of a MS protocol with letrozole plus hMG in non-obese infertile women with PCOS candidates to IVF/ICSI.

## Methods

### Patients

Among all infertile women with PCOS referred to our Reproductive Unit for couple infertility from January 2014 to December 2017, we retrospectively evaluated those who satisfied the following entry criteria: having received a MS protocol with letrozole plus hMG or a CS with hMG alone in a GnRH antagonist co-treated IVF/ICSI cycle, and having received metformin in the preceding three to six months (see below). Exclusion criteria were age > 40 years, BMI > 26 kg/cm^2^, thyroid dysfunction, pituitary tumors, congenital adrenal hyperplasia, adrenal tumors, Cushing syndrome, androgen-secreting tumors, moderate-to-severe endometriosis. Diagnosis of PCOS was based on the NIH 2012 extension of ESHRE/ASRM 2003 criteria [10].

12 patients in MS group (15%) and 6 (13,3%) in the CS group had an history of IUI cycles (1–3 cycles). None had a previous IVF cycle. Indication to IVF are displayed in Table 1. Since the IUI cycles were performed in other centers before metformin pre-treatment, we did not take into account the previous response to gonadotropin treatment when setting the FSH starting dose.

**Table 1:** Comparison of clinical parameters in patients stratified in two groups according to stimulation protocol (letrozole plus HMG or HMG-only)

	Group 1 (Let-HMG)	Group 2 (HMG)	*p*-value*
N	80	45	/
Age (years)	34.5 (31–37) [24–40]	34 (31.5–36.5) [27–40]	0.959
Male factor infertility (%)	53.7	55.5	0.84
Endometriosis (%)	5	4.4	0.88
Tubal factor (%)	6	4	0.63
BMI (kg/cm^2^)	21,9 (20–25) [16–31.5]	23 (20–24) [18–29]	0.736
Basal FSH (mIU/ml)	6.4 (5.3–7.4) [2–12]	6.4 (5.5–7.2) [4.5–9]	0.795
Basal LH (mIU/ml)	5.2 (4.15–6.7) [0.88–11]	4.7 (3.8–6.9) [2.55–11]	0.402
Basal E2 (pg/ml)	42.7 (30.2–59.9) [13–238]	43 (36–51) [13–91]	0.977
Antral follicle count (n)	12 (10–16) [7–27]	12 (10–14) [9–20]	0.316
AMH (ng/ml)	3.5 (2.4–4.9) [1.16–12.3]	4 (2.5–6.7) [1.2–10]	0.222

Data are displayed as median with upper and lower range in round parentheses and ranges in squared parentheses

*Two-tailed Mann-Whitney test

Before being enrolled for the IVF/ICSI program, all patients received a three to six months pre-treatment with metformin extended release (Glucophage Unidie, Bruno Farmaceutici Italy) 1200–2000 mg daily, in order to reduce the risk of ovarian hyperstimulation syndrome (OHSS) and to suppress the excessive ovarian production of androgens [11]. Obese patients were invited to lose weight by following a hypocaloric low glycemic index diet and exercise; patients whose BMI remained higher than 26 were excluded by the study.

In order to assess eligibility to IVF/ICSI, all patients underwent a careful physical examination, including height and weight measurement. Antral follicle count (AFC) was evaluated in the early follicular phase (day 2–5) by transvaginal ultrasound. Blood tests were performed on day 2 to assess basal FSH, LH, Estradiol (E2) and antimullerian hormone (AMH) serum levels. Endometrial thickness was evaluated by ultrasound.

### Ovarian stimulation protocol and study design

125 patients that met the entry criteria were divided in two groups according to the received stimulation protocol: group 1 comprised 80 patients who received Letrozole (Ramates, Just Pharma, Rome, Italy) 2,5 mg daily starting from day 2 through day 6 of the menstrual cycle, plus hMG (Meropur, Ferring Italy) administered at a fixed-dose of 150 IU starting from day 3 to day 6 with further dose adjustments, while group 2 patients comprised 45 women who underwent an individualized, tailored hMG dose regimen starting from day 2 onwards, the starting dose being set according to a nomogram based on patients age, AFC and AMH [12]. The resulting mean ± SD starting dose was 182,22 IU/die ± 14,73 (range 100–300) IU/die. Allocation to treatment groups was determined by patients willingness to receive letrozole: some general practitioners dissuaded their patients from taking letrozole due to the recommendation against its use in premenopausal women provided in the drug label. All patients from group 1 gave written informed consent to the off-label use of letrozole in IVF.

In both groups a GnRH antagonist, Ganirelix acetate (Orgalutran, Merck Sharp & Dohme, UK), was started once the leading follicle(s) reached 14 mm in diameter. Serial ultrasound examination and evaluation of serum E2 levels were used to assess follicular maturation and endometrial thickness. Human chorionic gonadotropin (hCG) 7.500 to 10.000 IU s.c. was administered when at least two follicles reached a mean diameters of 18 mm. Progesterone level was assessed at hCG day.

IVF or ICSI was performed as clinically appropriate, with embryo transfer performed either in day 3 or in day 5, since no superiority of blastocyst compared to cleavage-state embryo transfer has been demonstrated [13]. Patients were eligible for frozen embryo transfer when serum progesterone (P) level was higher than 1.75 ng/ml [14] and/or E2 level was higher than 3500 pg/ml on hCG day. Luteal phase was supported by vaginal micronized progesterone 600 mg/day (Progeffik, Effik Italy).

### Statistical analysis

Since the observed variables did not follow a normal distribution according to one-sample Kolmogorov-Smirnov test, the differences in clinical parameters and in IVF outcome in patients stratified in two groups according to the stimulation protocol were evaluated by two-sided Mann Whitney U test for continuous variables, by chi square for categorical variables, and by z-test for proportions.

Statistical significance was set at *p* < 0.05 for all analyses. All computations were performed using SPSS for Windows.

### IRB approval

The present study was approved by the local IRB (IEC Azienda Ospedaliera Universitaria Policlinico di Bari, register number 4990, April 5, 2016).

## Results

Patients clinical parameters are summarized in Table 1. Both groups were comparable for age, BMI and ovarian reserve markers.

**Fig. 1:**
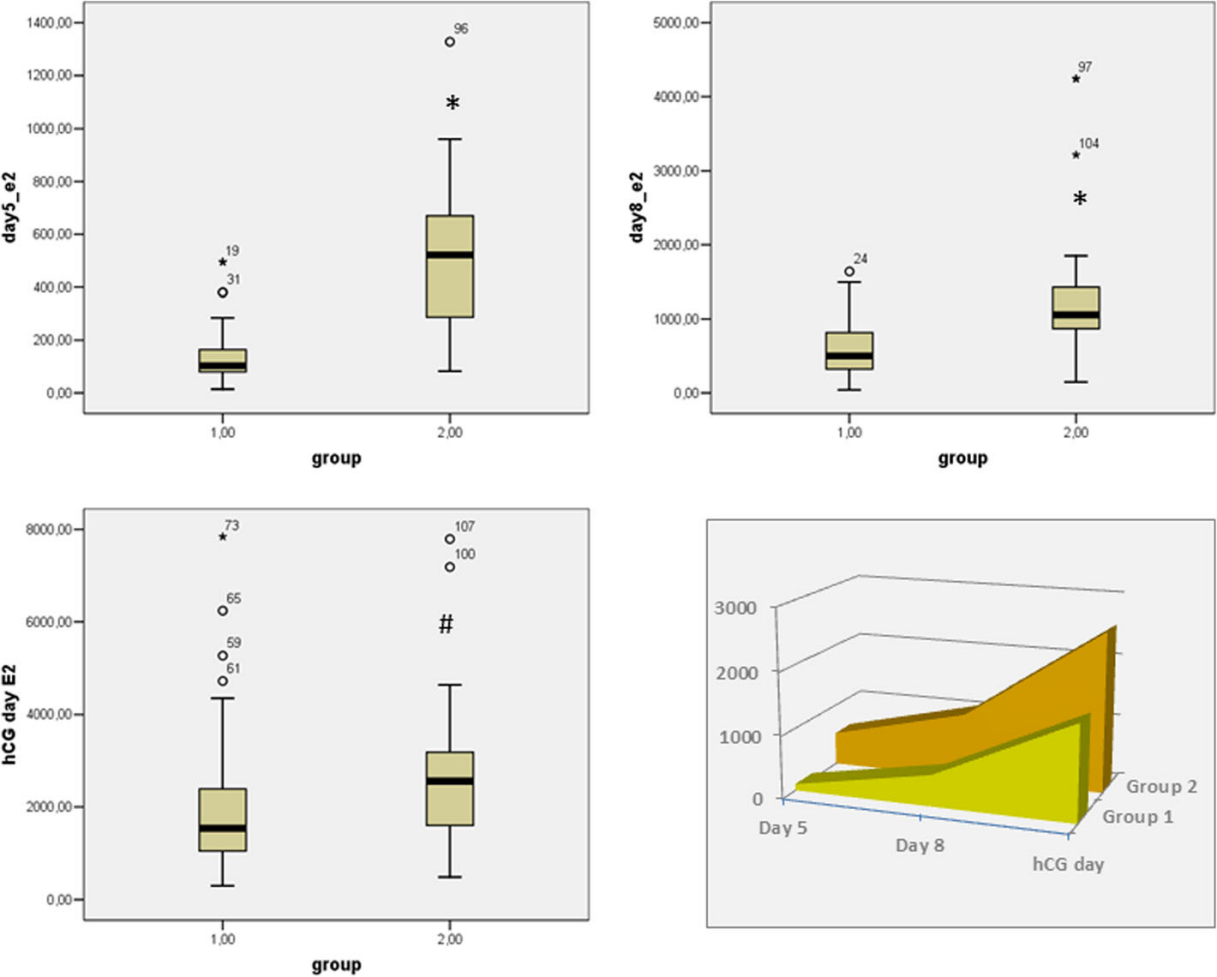
Serum estradiol levels during ovarian stimulation protocol in patients with PCOS receiving letrozole plus fixed-dose hMG (Group 1) or tailored dose hMG (Group 2). A – C: Box plot displaying the estradiol levels in stimulation day 5 (A), 8 (B) and at hCG day (C). D: area graph displaying the dynamic of estradiol levels rise during ovarian stimulation in the two groups of patients. * = *p* < 0.0001

Table 2 display the stimulation and IVF outcome variables obtained to both group of patients. Group 1 patients required a significantly lower amount of gonadotropins units, and showed significantly lower day 5, day 8 and hCG day E2 levels compared to group 2 (Fig. 1). This difference in E2 level, however, did not affect the number of maturing follicles (Table 3), which were comparable among groups.

**Table 2:** Stimulation and IVF outcome variables of PCOS patients under letrozole plus HMG or HMG-only stimulation protocol

	Group 1 (Let-HMG)	Group 2 (HMG)	*p*-value
Days of stimulation (N)	11 (10–12) [8–15]	10 (10–12) [9–15]	0.246*
HMG dose (units)	1350 (1200–1500) [626–2475]	1800 (1337–3110) [1050–5325]	< 0.0001*^p^
E2 day 5 (pg/ml)	103 (73–182) [14–495]	525 (284–703) [82–1328]	< 0.0001*^p^
E2 day 8 (pg/ml)	502 (322–826) [39–1639]	1056 (829–1439) [147–4244]	< 0.0001*^p^
E2 hcg day (pg/ml)	1538 (1043–2391) [300–7845]	2561 (1600–3238) [482–7793]	0.001*
Progesterone at hCG day (ng/ml)	0.8 (0.6–1.1) [0.17–2.5]	0.86 (0.6–1.37) [0.2–4.9]	0.183*
Endometrial thickness (mm)	9.5 (8.6–10.7) [6.7–13]	10 (8.7–10.9) [7.3–15.3]	0.25*
N. oocytes retrieved	6 (5–8) [3–17]	6 (5–9) [3–17]	0.361*
N. MII oocytes	5 (4–7) [2–12]	5 (4–7) [2–14]	0.593*
hCG day E2/follicles > 16 mm ratio	193 (128–292) [28–569]	320 (219–434) [87–974]	< 0.0001^p^
hCG day E2/total oocyte ratio	233 (169–329([53–590]	372 (309–660) [116–1025]	0.001^p^
hCG E2/MII oocytes ratio	313 (221–430) [60–1077]	473 (309–660) [120–1025]	0.001^p^
N. embryos	3 (2–4) [1–12]	3 (2–3) [1–9]	0.895*
N. good quality embryos	2 (2–3) [0–8]	2 (1.5–3) [0–5]	0.864*
N. transferred embryos	2 (2–3) [1–3]	2 (2–3) [0–3]	0.605*
N. good quality embryos transferred	2 (1–2) [0–3)	2 (1–2) [0–3]	0.815*
N. fresh cycles (%)	80	60	0.016^#^
Fertilization rate (%)	78.9	79.9	0.941^#^
Pregnancy rate in fresh cycles N (%)	33/64 (51)	10/36 (27.7)	0.021^§^
Clinical pregnancy rate in fresh cycles N (%)	30/64 (46.8)	10/36 (27.7)	0.061^§^
Cumulative pregnancy rates (fresh + cryo) N (%)	36/80 (48.7)	12/45 (26.6)	0.016^§p^
Cumulative clinical pregnancy rate N(%)	34/80 (42.5)	11/45 (24.4)	0.044^§^

Data are displayed as median with upper and lower range in round parentheses and ranges in squared parentheses, or as count with percentages in parentheses

*Two-tailed Mann-Whitney test

^#^2-sided Pearson Chi-square test

^§^Z-test for two independent proportions

^p^the study is powered to detect this difference

**Table 3:** Ovarian follicles behavior under letrozole plus HMG or HMG-only stimulation protocol

Parameter	GROUP 1 (LET-HMG)	GROUP 2 (HMG)	*p*-value*
Day 5 follicles< 10 mm	9 (5.5–11) [0–21]	9 (8–13) [4–20]	0.097
Day 8 follicles < 10 mm	4 (2–7) [0–13]	5 (4–8) [2–18]	0.031^p^
hCG day follicles< 10 mm	3 (0–4) [0–11]	4 (2–4.7) [0–13]	0.024^p^
Day 5 follicles > 11 mm	3 (1–5.5) [0–20]	2 (1–4) [0–8]	0.131
Day 8 follicles > 13 mm	6 (5–8) [0–23]	5 (2–7) [1–20]	0.009^p^
hCG day follicles > 16 mm	7 (6–9) [3–25]	7 (6–9) [3–15]	0.836

Data are displayed as median with upper and lower range in round parentheses and ranges in squared parentheses

*Two-tailed Mann-Whitney test

^p^the study is powered to detect this difference

The use of letrozole did not impact on progesterone levels (at hCG day) and on endometrial thickness, both resulting comparable among groups; on the other hand, MS with letrozole resulted in an higher percentage of fresh cycles compared to the CS protocol (80 vs 60%, *p* = 0.016). In the MS group, the freeze-all policy was applied to 16 patients (20%): in 9 cases for elevated E2 levels, in 5 cases for P rise, in 2 patients for both factors. In the CS group, the freeze-all strategy was applied to 18 patients (40%): 11 cases for high E2 levels, in 4 cases for P rise, in 3 cases for both reasons. 6 patients (7%) in MS group and 12 (26,6%) in the CS group (*p*-value = 0 .0036) received a GnRH agonist trigger (0,3 mg of triptorelin) to prevent OHSS. No patient from both groups needed hospitalization for OHSS.

The oocyte yield, in terms of total and MII oocytes number, did not differ among groups; the number of total and good quality embryos obtained was comparable as well in the two groups.

The clinical pregnancy rate (CPR) in fresh transfers did not differ among groups; only a trend for higher cumulative CPR was observed in group 1 compared to group 2 (critical z 1.9599640, 1-β 0.55).

No side effects were reported by patients who received Letrozole.

## Discussion

The results of this pilot study suggest that a mild stimulation protocol with Letrozole may represent an effective alternative to standard IVF stimulation protocols for non-obese PCOS women pre-treated with metformin, as it provides a comparable IVF outcome to the standard GnRH antagonist protocol, with the advantage of reducing the FSH dose, lowering the estradiol levels under stimulation, and reducing the need of embryo cryopreservation to prevent OHSS.

It has been proposed that the major advantage of using MS protocols is to allow the development of the healthier follicles with more competent eggs, given that higher gonadotropins dose may disrupt the physiologic hormonal milieu inside the follicles [15]. Indeed, earlier studies suggest that CS protocols may have a negative impact on the developmental and implantation potential of human embryos [reviewed in 2]. These results were challenged by other studies demonstrating that the degree of exposure to exogenous gonadotropins did not significantly modify the likelihood of blastocyst aneuploidy in patients with a normal ovarian response to stimulation [16]; relevantly, those earlier results were obtained from the analysis of cleavage stage embryos, so that the embryos aneuploidy rates were artificially increased by the inclusion of mosaic embryos with likely innate abilities to self-correct downstream beyond blastocyst stage (reviewed in [17]).

It has to be highlighted that the patients enrolled in the present study displayed lower than expected AFC and AMH values. The median AFC found in our patients was comparable, while the median AMH was slightly higher, than that of an age-matched cohort of 5705 women comprising poor, normal and hyperresponders, [18]. It has to be pointed out, however, that AMH and AFC values were obtained at the end of metformin treatment and before the start of the stimulation protocols: since metformin has been found to significantly reduce both AMH and AFC in PCOS patients [19] we hypothesize that the resulting AFC and AMH values are the consequence of metformin treatment. The mechanism trough which metformin reduces AFC and AMH hasn’t been fully clarified to date, although the demonstrated effect on metformin in reducing the number of primary, preantral and atretic follicles while increasing that of antral follicles by angiogenic factors levels restoration [20] could explain this finding. Also the low number of oocytes obtained in both groups may be explained by the lower AFC and AMH values than expected in PCOS patients. Still the number of MII oocytes was extremely variable in both groups, ranging from 2 to 12 and from 2 to 14 in both groups respectively (Table 2), this variability being probably due to the wide age range of patients enrolled in the study (24–40 and 27–40 respectively, Table 1).

MS protocols may also improve/optimize the endometrial receptivity by lowering the estradiol levels [15]. It has been demonstrated that supraphysiologic estradiol levels may impact on the endometrial receptivity [21, 22]. Recent large-sample studies has even found that supraphysiologic estradiol levels may expose the offspring to an higher risk of low birth weight [23–25].

Although MS cannot be used as first line protocol for every patient [8, 17], the present study is suggestive for its preferable employment in non-obese PCOS patients pretreated with metformin, as it provided a comparable IVF outcome to CS despite significantly lower estradiol-to-follicles and estradiol-to-MII oocytes ratios (Table 2), suggesting that higher estradiol levels are unneeded when letrozole is employed. The reason for this finding could be explained by the dynamics of androgen secretion following letrozole administration.

Letrozole plays its role by inhibiting the cytochrome P450 isoenzymes 2A6 and 2C19 of the aromatase enzyme complex, resulting in a release of the hypothalamic–pituitary axis from estrogenic negative feedback and, consequently, in stronger GnRH pulses and FSH secretion rise, with the consequential stimulating effect on the growth of ovarian follicles [26]. In addition, the increase of intra-ovarian androgens**,** secondary to aromatase inhibition**,** augments the follicular sensitivity to FSH and enhances the early follicular growth. Several studies have demonstrated a direct relationship between androgen receptor (AR) mRNA and FSH receptor (FSHr) in granulosa cells, as well as the priming effect of androgens on FSHr expression modulated by the enhanced AR expression (reviewed in [27]). The relationship between AR and FSHr in granulosa cells is amplified in PCOS patients [28]. Moreover, androgens have been found to attenuate follicular atresia through nuclear and extranuclear signaling pathway by enhancing the expression of the microRNA miR-125b [29]. When Letrozole is administered in the early follicular phase, an increased expression of FSHrs together with an higher FSH release from the pituitary is expected, resulting in increased androgen pool which prevent follicular atresia. High androgen levels could, therefore, promote follicular growth in the early follicular phase in a way that could be independent, to a certain extent, from estradiol level. Indeed, at that stage the estradiol levels were very low in letrozole-treated patients compared to controls (Table 2). Interestingly, it has been demonstrated that lower estradiol levels in the early follicular phase don’t affect the follicular development [30]. Conversely, when follicles growth proceeds, the role of androgens in follicular growth begins negligible; indeed, a lower expression of the AR gene in human GC in the large pre-ovulatory follicles compared to small ones have been reported [28, 31].

Another potentially beneficial effect of using the present MS protocol for IVF in non-obese PCOS women is the lower need to proceed with embryo cryopreservation to prevent OHSS compared to controls. It has been suggested that reducing the need of frozen embryo transfer could have a beneficial impact on the perinatal outcome of IVF singletons [32, 33], as embryo cryopreservation may promote alteration of DNA methylation reprogramming enzymes [34].

Letrozole is still contraindicated in premenopausal women due to the results of a follow up study, presented at the 2005 ASRM meeting but never published on a peer reviewed journal, on 150 babies born to women who took letrozole for ovulation induction, showing that cardiac and locomotor anomalies were overrepresented in the letrozole babies compared with the control group of normal fertile women Indeed, a further multicentre study demonstrated that the major malformation rate was lower in letrozole group compared to clomiphene citrate group, with the incidence of cardiac anomalies being seven times lower compared to clomiphene [35]. Recently, letrozole has been even found to significantly decrease the risk of miscarriage, without increasing the risk of major congenital anomalies or adverse pregnancy or neonatal outcomes, when compared with natural cycles in patients undergoing ART [36]. Still, as no therapeutic indication has been listed for ovulation induction or IVF in the letrozole package information, and the recommendation against its use in premenopausal women persists, obtaining IRB approvals for clinical trials aiming at evaluating the use of letrozole in MS-IVF protocols remains challenging.

The present study has some limitations. Data have been retrospectively evaluated, however since all data were stored in a database management system being in use to the IVF unit, no data was missed. The study sample size, although large enough to reject the null hypothesis in some comparisons (see Tables 2-3), is too small to allow generalization of our findings with regards to the IVF outcome. It has to be highlighted, furthermore, that the results of the present study may apply only to non-obese PCOS women pre-treated with metformin for at least three months, in view of the lower than expected AFC and AMH values observed in our patients.

## Conclusions

In conclusion, the present study suggests that the use of letrozole as adjuvant treatment to mild stimulation protocols for IVF may be a safe and cost-effective an effective alternative to standard ovarian stimulation protocols for non-obese PCOS patients undergoing metformin pre-treatment, as it provides comparable IVF outcome without requiring high FSH dose, and avoids supraphysiological estradiol levels, which may be detrimental to the developmental and implantation potential of embryos.

## Abbreviations

AFC: Antral follicle count
AMH: Anti mullerian hormone
CS: Conventional IVF stimulation protocols
E2: Estradiol
GnRH: Gonadotropin releasing hormone
hCG: Human chorionic gonadotropin
HMG: Human menopausal gonadotropin
ICSI: Intracytoplasmic sperm injection
IVF: In vitro fertilization
MII: Metaphase II
MS: Mild stimulation
OHSS: Ovarian hyperstimulation syndrome
PCOS: Polycystic ovarian syndrome
